# Identification of Sertoli cell-specific transcripts in the mouse testis and the role of FSH and androgen in the control of Sertoli cell activity

**DOI:** 10.1186/s12864-017-4357-3

**Published:** 2017-12-15

**Authors:** U. Soffientini, D. Rebourcet, M. H. Abel, S. Lee, G. Hamilton, P. A. Fowler, L. B. Smith, P. J. O’Shaughnessy

**Affiliations:** 1 Institute of Biodiversity, Animal Health & Comparative Medicine, College of Medical, Veterinary and Life Sciences, University of Glasgow, G61 1QH, Glasgow, UK; 2 MRC Centre for Reproductive Health, University of Edinburgh, The Queen’s Medical Research Institute, 47 Little France Crescent, Edinburgh, EH16 4TJ UK; 3 Department of Physiology, Anatomy and Genetics, University of Oxford, Le Gros Clarke Building, Oxford, OX1 3QX UK; 4 Glasgow Polyomics, College of Medical, Veterinary and Life Sciences, University of Glasgow, G61 1QH, Glasgow, UK; 5 Institute of Medical Sciences, School of Medicine, Medical Sciences & Nutrition, University of Aberdeen, Foresterhill, Aberdeen, AB25 2ZD UK; 6 School of Environmental and Life Sciences, University of Newcastle, Callaghan, Newcastle, 2308 Australia

**Keywords:** Sertoli, RNAseq, Microarray, Transcriptome, Germ cell, Testis, Follicle-stimulating hormone, Androgen

## Abstract

**Background:**

The Sertoli cells act to induce testis differentiation and subsequent development in fetal and post-natal life which makes them key to an understanding of testis biology. As a major step towards characterisation of factors involved in Sertoli cell function we have identified Sertoli cell-specific transcripts in the mouse testis and have used the data to identify Sertoli cell-specific transcripts altered in mice lacking follicle-stimulating hormone receptors (FSHRKO) and/or androgen receptors (AR) in the Sertoli cells (SCARKO).

**Results:**

Adult iDTR mice were injected with busulfan to ablate the germ cells and 50 days later they were treated with diphtheria toxin (DTX) to ablate the Sertoli cells. RNAseq carried out on testes from control, busulfan-treated and busulfan + DTX-treated mice identified 701 Sertoli-specific transcripts and 4302 germ cell-specific transcripts. This data was mapped against results from microarrays using testicular mRNA from 20 day-old FSHRKO, SCARKO and FSHRKO.SCARKO mice. Results show that of the 534 Sertoli cell-specific transcripts present on the gene chips, 85% were altered in the FSHRKO mice and 94% in the SCARKO mice (mostly reduced in both cases). In the FSHRKO.SCARKO mice additive or synergistic effects were seen for most transcripts. Age-dependent studies on a selected number of Sertoli cell-specific transcripts, showed that the marked effects in the FSHRKO at 20 days had largely disappeared by adulthood although synergistic effects of FSHR and AR knockout were seen.

**Conclusions:**

These studies have identified the Sertoli cell-specific transcriptome in the mouse testis and have shown that most genes in the transcriptome are FSH- and androgen-dependent at puberty although the importance of FSH diminishes towards adulthood.

**Electronic supplementary material:**

The online version of this article (10.1186/s12864-017-4357-3) contains supplementary material, which is available to authorized users.

## Background

The development and function of the testis is critically dependent on the Sertoli cells. These cells initially differentiate from the coelomic epithelium, at around embryonic day 11.5 (e11.5) in the mouse, and act to induce formation of the seminiferous tubules and the fetal Leydig cell population [1]. Post-natally, the Sertoli cells are essential for spermatogenesis and cell ablation studies have shown that the Sertoli cells are also required for development and maintenance of both the adult Leydig cells and peritubular myoid cells and for normal development of the testicular vasculature [2–4]. Some specific pathways involved in these cell-cell interactions have been identified, such as the role of DHH in Leydig cell development [5, 6] but, in most cases, the cellular and molecular mechanisms by which the Sertoli cells exert control over testis development and function are unknown. Given the central role of the Sertoli cell in testis biology, identification of these pathways has to be a high priority for current research, particularly with respect to androgen production, ageing and adult male health [7, 8]. A step towards this goal would be identification of Sertoli cell-specific transcripts – ie those testicular transcripts that are only expressed in the Sertoli cells and are likely, therefore, to be involved in Sertoli-specific functions. Previously, we have shown that cell ablation can be used to identify cell-specific transcripts [9] and so we have now used the recently described diphtheria toxin (DTX) model of Sertoli cell ablation [3] to identify Sertoli cell-specific transcripts using RNAseq. As previously [9], one confounding factor in identifying cell-specific transcript expression through cell ablation studies in the testis is the loss of germ cells coincident with Sertoli cell ablation [2]. To circumvent this problem, we have used busulfan to generate a germ cell-free mouse prior to Sertoli cell ablation [9]. This also has the added advantage that comparison between normal mice and busulfan-treated animals allows identification of germ cell-specific transcripts.

During post-natal development, and in the adult, the Sertoli cells are regulated by follicle stimulating hormone (FSH) and androgen [10]. In the absence of FSH, or the FSH-receptor (FSHR), Sertoli cell number and germ cell number is reduced although the animals are fertile [11, 12]. In animals lacking androgen receptors (AR) specifically on the Sertoli cells (SCARKO) the Sertoli cell number is normal but spermatogenesis is blocked at meiosis and the animals are infertile [13]. Double knockout of both the FSHR and AR in the Sertoli cells has a synergistic effect leading to a marked failure in germ cell development and spermatogenesis [14]. There have been a number of studies which have examined global changes in transcript levels in ARKO and SCARKO mice but there has been no strong consensus about the transcripts affected in these animals [15]. This may be partly because these studies are confounded by changes in germ cell-specific transcript levels as a result of the disruption to spermatogenesis. There have, to date, been no studies of changes in overall transcript levels in FSHRKO mice or in combined FSHRKO.SCARKO mice. Identification of Sertoli cell-specific genes affected by FSHR, AR and FSHR + AR knockout would be an important step forward in understanding how FSH and androgen regulate the Sertoli cells and, in turn, how the Sertoli cells regulate testis function. We have therefore carried out microarray studies on control, FSHRKO, SCARKO and FSHRKO.SCARKO mice and integrated the data with the cohort of Sertoli cell-specific genes identified by cell ablation. This has allowed is to identify Sertoli cell specific transcripts altered in the different mouse models.

## Methods

### Animals and treatments

All animal maintenance and handling followed U.K. Home Office regulations and all animal procedures were carried out under U.K. Home Office License and with the approval of local ethical review committees at the Universities of Glasgow, Edinburgh and Oxford. FSHRKO, SCARKO and FSHRKO.SCARKO mice were generated at the University of Oxford as published previously [16] from mice obtained originally from Prof G Verhoeven and Dr. K De Gendt (Catholic University of Leuven, Belgium) and from mouse colonies bred at the University of Oxford [16]. Mice were killed at 1d, 5d, 20d and 8 weeks of age and testes stored in liquid N_2_ until used for microarray studies or qPCR. Amh-Cre;iDTR mice (referred to as iDTR mice), which carry the diphtheria toxin receptor (DTR) specifically in the Sertoli cells, were generated as described previously [2, 3] from mice originally obtained from Professor A Waisman (through the Jackson Laboratories, Maine, USA) and from Professor F Guillou (Université de Tours, France). Adult iDTR mice (>120 days) were injected once with busulfan (30 mg/Kg) or vehicle [2, 17] and 50 days later they were injected with DTX (100 ng) or vehicle. Animals were killed 7 days later and one testis from each animal was frozen and stored in liquid N_2_. To confirm identification of Sertoli cell transcripts from RNAseq data, mRNA was used from a cohort of adult iDTR mice described previously [2]. These animals were injected once with DTX only (without busulfan treatment) and were killed 7d, 30d, 90d or 1 year later. Similarly, to confirm identification of germ cell transcripts from RNAseq data, mRNA was used from control mice (purchased from Harlan UK, Bicester, UK) treated once with busulfan and killed at different times up to 50 days later [17]. Finally, control, untreated mice from a different colony (bred at the University of Glasgow) [18] were used to examine normal development of transcript expression.

### RNAseq

RNA was extracted from whole testes of individual animals using RNAeasy kits with on-column DNAse treatment (Qiagen Ltd., Manchester, UK). Polyadenylated RNA Sequence libraries were constructed using the Illumina TruSeq Stranded mRNA sample prep kit (Illumina, Cambridge, UK). The library was single-end sequenced on an Illumina NextSeq 500 with the NextSeq 500/550 High Output kit version 2 for 75 sequence cycles. Sequencing depth averaged 43.7 million reads per sample (range 31.6–61.2). The sequence reads were filtered for low quality bases and contaminating adapter sequences using Cutadapt [19] (version 1.5). The quality-trimmed reads were aligned to the mouse genome (version GRCm38.p4) using HiSat [20] (version 0.1.2-beta) and mean alignment per sample was 96.9% (range 96.1–97.5%). Differential expression analysis was performed using Cuffdiff [21] for the Cufflinks package [22]. The bioconductor package cummeRbund [23] was used to investigate the output from the Cuffdiff package. Identification of signal peptides in transcripts of interest was carried out by manual curation of relevant NCBI GenBank files (https://www.ncbi.nlm.nih.gov/genbank/) while secreted proteins were identified through comparison with a database downloaded from the Human Protein Atlas (https://www.proteinatlas.org/search/protein_class:Predicted%20secreted%20proteins).

### Microarrays

Microarrays were carried out using RNA extracted from whole testes of 20-day old FSHRKO, SCARKO, FSHRKO.SCARKO and control mice. RNA from testes of individual animals was extracted using RNeasy kits (Qiagen Ltd., Manchester, UK). Samples of total RNA (8 μg) from individual animals (4 per group) were reverse transcribed and then in vitro transcribed and hybridised to mouse MOE430A arrays (Affymetrix, Santa Clara, CA, USA) according to the GeneChip expression technical manual (Affymetrix, Santa Clara, CA, USA) as previously reported [24]. Gene transcript levels were determined from data image files using algorithms in Gene Chip Operating Software (GCOS1.2, Affymetrix). Data were normalised to testis volume and Sertoli cell number as described in Results (Additional file 1). Array data and RNAseq data were aligned using a relational database (Access, Microsoft Corp, Washington, USA) using the gene symbols to connect the databases. This allowed Sertoli cell-specific transcripts on the arrays to be identified.

### Real-time PCR

Testis RNA was extracted using Trizol (Life Technologies, Paisley, UK) and levels of specific mRNA species were measured by real-time PCR. Total RNA was reverse transcribed using random hexamers and Moloney murine leukaemia virus reverse transcriptase (Superscript III, Fisher Scientific UK Ltd., Loughborough, UK) as described previously [25]. To normalise the data, external standard (luciferase mRNA: Promega UK, Southampton, UK) was added to each testis at the start of the RNA extraction [26]. For real-time PCR the SYBR green method was used with SYBR mastermix (Agilent Technologies, Wokingham, UK) [27]. All primers were designed by Primer Express 2.0 (Applied Biosystems, Warrington, UK) or Primer-BLAST (https://blast.ncbi.nlm.nih.gov/Blast.cgi) using parameters previously described [28] and the primers used are described in Additional file 2. Transcript levels were normalised to the luciferase external standard, which generates a relative value of transcript expression per testis [29].

### Statistical analysis

Data from the arrays were analyzed using two-factor analysis of variance (ANOVA) with each gene knock-out (FSHRKO or SCARKO) as one of the factors. Where the interaction between the factors was significant, this means that the effect of the double knockout (FSHRKO.SCARKO) was not simply an additive effect of each gene knockout alone. Where there was no significant interaction *P* values were taken directly from the ANOVA. Where the interaction was significant, post-hoc testing was used to calculate P values for FSHRKO and SCARKO groups. The significance of differences between groups in the RNAseq were calculated by t-test or single factor ANOVA. In all cases, differences between groups were considered significant with *P* < 0.05 and with the false discovery rate (FDR) = 0.05 [30]. Real-time PCR data was analysed using single factor or two-factor ANOVA.

## Results

### Effects of busulfan and DTX on the testis transcriptome

RNAseq analysis of testis transcript expression in control, busulfan-treated and busulfan + DTX-treated mice identified 25,255 transcripts which were detectable in at least one group (Additional file 3). Treatment of iDTR mice with busulfan caused a significant (P < 0.05, FDR = 0.05) change in the expression of 18,095 transcripts compared to control (6961 transcripts increased, 11,134 transcripts decreased; Additional file 4) while treatment with busulfan followed by DTX caused a significant change in levels of 5280 transcripts *compared to busulfan alone* (2891 transcripts increased and 2389 transcripts decreased; Additional file 4). Since treatments with busulfan and with DTX will specifically target germ cells and Sertoli cells respectively (with close to 100% efficiency) then transcripts markedly reduced following each treatment regime are highly likely to be germ cell- or Sertoli cell-specific. The only probable exceptions will be transcripts in another cell type which are highly dependent on the germ cells or Sertoli cells and which may be wrongly categorised as germ cell- or Sertoli cell-specific if their expression drops markedly after cell ablation. This is discussed further below but we would not anticipate numbers of such transcripts to be large. Transcripts in the RNAseq database that show a less-marked response to cell ablation (eg 30–70% decrease) are likely to be expressed in more than one cell type and so data in Additional file 4 should be treated with caution and should be cross-referenced to Additional file 3.

**Fig. 1:**
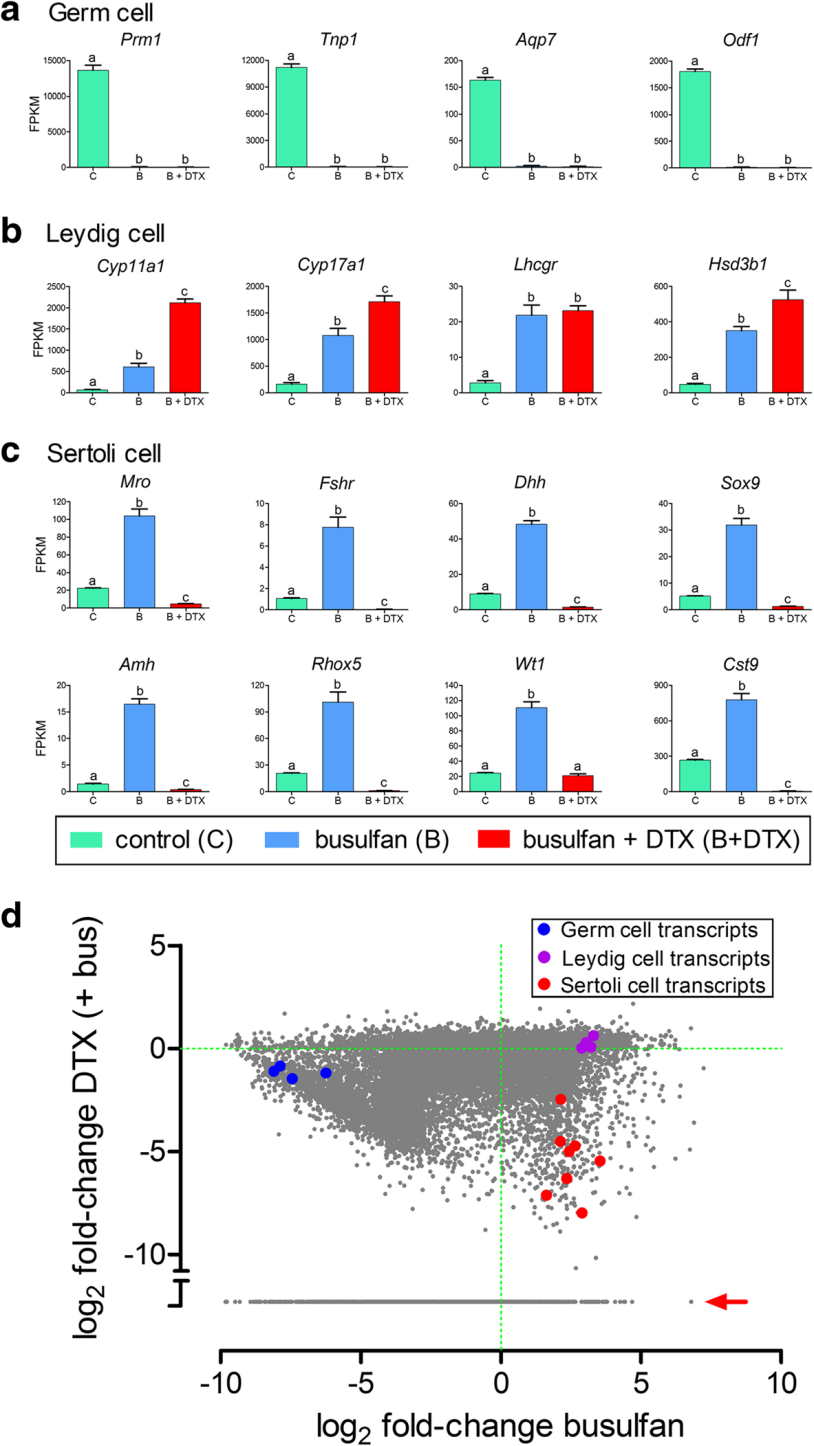
Data from RNAseq study showing transcript expression in control, busufan-treated and busulfan + DTX-treated mice. Data shows expression of known germ-cell transcripts (**a**), Leydig cell transcripts (**b**) and Sertoli cell transcripts (**c**). Results show mean ± SEM of 4 or 5 animals per group. In (**d**) data from all transcripts detected in the RNAseq study is shown as the transcript number log ratio of DTX + busulfan/ busulfan alone (log_2_ fold-change DTX (+bus)) plotted against the log ratio of busulfan alone/control (log_2_ fold-change busufan). Known germ cell transcripts from a) are shown in blue, Leydig cell transcripts from b) are shown in purple and Sertoli cell transcripts from c) are shown in red. When transcript expression in the DTX + busulfan group was zero the log_2_ fold-change DTX (+bus) could not be calculated and the points are indicated by a red arrow. In a) to c) groups with different letter superscripts are significantly (*P* < 0.05) different

As a proof of concept that cell ablation will identify cell-specific transcripts, transcript data (from the RNAseq study) for a number of known germ cell-, Leydig cell- and Sertoli cell-specific genes are shown in Fig. 1a-c. The known germ cell-specific genes showed the expected marked reduction in transcript numbers following busulfan treatment (Fig. 1a). Leydig cell-specific genes showed a significant increase in transcript levels after busulfan treatment and in some cases a further increase after DTX (Fig. 1b). The increase in transcript levels after busulfan is due largely to relative enrichment of somatic cells in the testis after germ cell ablation and the same effect is seen with Sertoli cell specific genes (Fig. 1c). This also explains the number of transcripts significantly increased after busulfan or DTX treatment (Additional file 4). The average enrichment factor after busulfan treatment, measured from 25 known somatic genes, was 6.49-fold although there was a wide range (2.92–11.94 fold). The wide range probably results from effects of germ cell depletion on somatic cell gene expression [17]. The increase in Leydig cell transcript number after DTX is due to further enrichment caused by Sertoli cell ablation and, in some cases, to a likely change in Leydig cell function following Sertoli cell ablation [2]. In contrast to Leydig cell transcripts, treatment with DTX led to an expected and marked decrease in Sertoli cell-specific transcript numbers (Fig. 1c). There was some variation in this response which is likely to be an indication that some known Sertoli cell transcripts (eg *Wt1*) are also expressed to a small extent in other somatic cells. Figure 1d is a scatter plot showing all transcripts from the RNAseq study expressed as fold-change in transcript level following busulfan (relative to control) vs fold change following busulfan + DTX (relative to busulfan alone). The fold changes in the 8 known Sertoli cell-specific genes (in red), 4 Leydig cell genes (in purple) and 4 germ cell genes (in blue), shown in Fig. 1a-c, are highlighted in Fig. 1d. The three sets of transcripts form distinct clusters on the diagram (Fig. 1d) with more spread apparent in the Sertoli cell cluster reflecting the variation seen in Fig. 1c.

### Identification of Sertoli cell-specific transcripts

From the data in Fig. 1d it is clear that different levels of stringency can be applied to the identification of Sertoli cell-specific genes. At the highest level of stringency, selecting only genes which show a greater than 5-fold increase in transcript number after busulfan followed by a 90% decrease in transcripts after DTX, identifies 114 transcripts. It is clear, however, that these cutoff limits will miss a significant portion of known Sertoli cell genes. We have, therefore, chosen less stringent criteria - more than 2 fold increase after busulfan and a 70% decrease (−3.3 fold) after DTX (and a significant effect of busulfan or busulfan + DTX) – to ensure that we identify most Sertoli cell-specific transcripts. This identifies 701 transcript species (Additional file 5) which fulfill the criteria and show a significant (*P* < 0.05, FDR = 0.05) reduction after DTX treatment. This list contains 495 protein-coding genes, 121 non-coding RNAs, 44 predicted pseudogenes and 41 other unclassified transcripts (Additional file 5). The disadvantage to using less stringent criteria is that transcripts decreased 70–80% after DTX are likely to be expressed at low levels in other testicular cells. Conversely, this is a list of transcripts highly enriched in the Sertoli cells which can be used to interrogate other databases and it is likely to include the overwhelming majority of Sertoli cell-specific transcripts. The data in Additional file 3 also allows investigators to apply more or less stringent criteria as necessary. Finally, the complete RNAseq dataset (Additional file 3) can be used to assess whether any gene of interest is likely to show Sertoli cell expression, even if it is expressed in more than one testicular cell type. For example, none of the platelet-derived growth factors or their receptors show any decrease in expression after DTX treatment suggesting there is little or no expression in adult mouse Sertoli cells [31]. Similarly, inhibin/activin subunits have been reported to be expressed in several cell types in the testis [32] and data in Additional file 3 indicates that *Inhbb* is primarily a Sertoli cell product in the adult testis while *Inhba* is primarily expressed in somatic cells but not the Sertoli cells. As a final example, there is some uncertainty about *Sox13* expression in the testis with spermatogonia, spermatocytes, Sertoli cells and Leydig cells suggested as possible sites [33–35]. Analysis of the RNAseq data shows that expression increases ~5-fold after busulfan and decreases about 30% after DTX. This would indicate that in the mouse Sox13 is not expressed in the germ cells, shows some expression in the Sertoli cells but is mostly expressed in another somatic cell type. Differences to other studies which used the rat [33, 34] may be due species-dependent variation in transcript localisation.

**Fig. 2:**
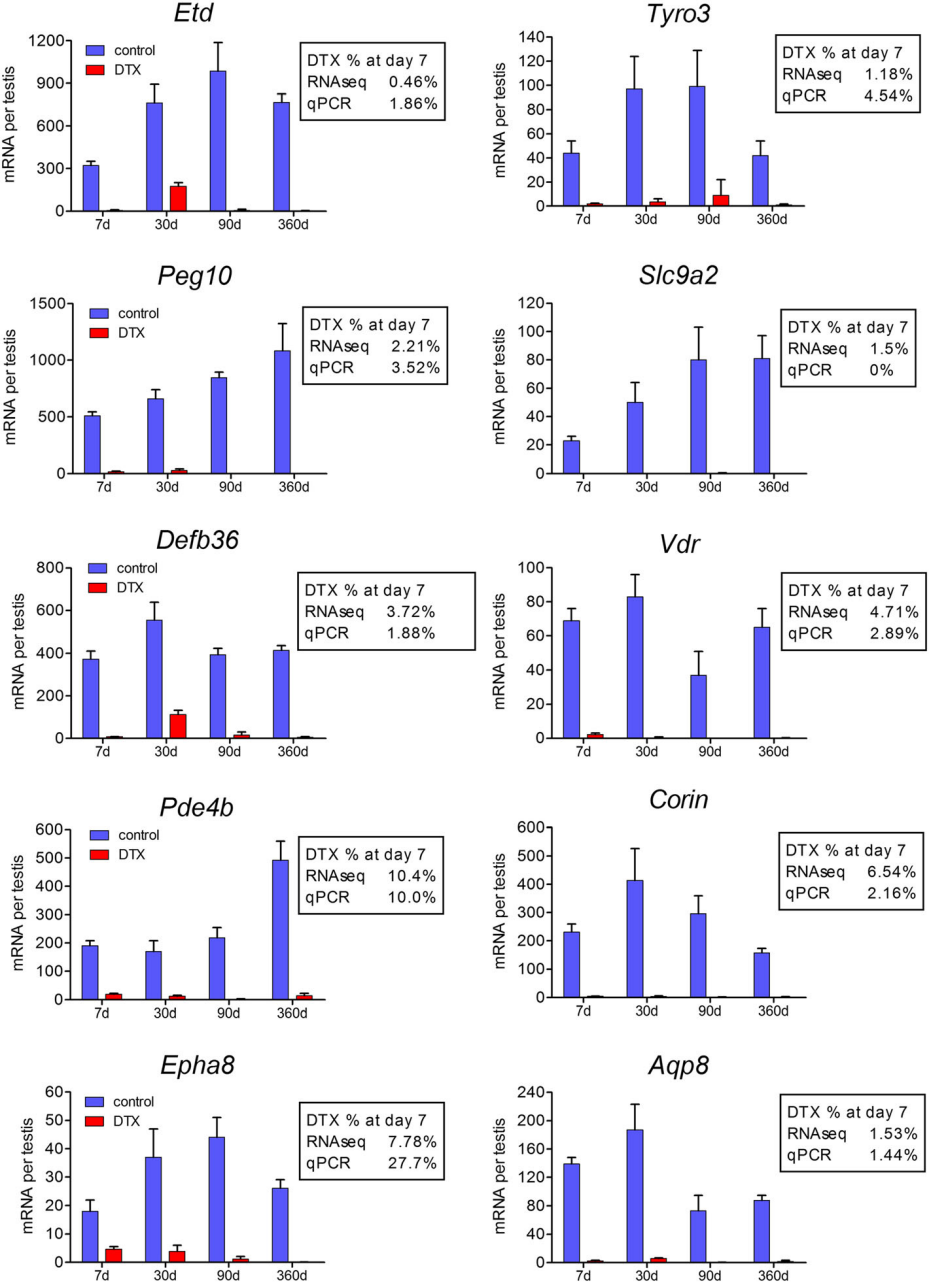
qPCR verification of selected RNAseq data. Ten transcripts were selected from the Sertoli cell specific group in Additional file 5 and expression was measured in iDTR mice 7, 30, 90 and 365 days after a single injection of DTX. The mean ± SEM is shown with 4–7 animals per group. For direct comparison, the transcript levels at day 7 (expressed as a percentage of control levels) are shown beside each graph for both RNAseq and qPCR data. Transcript species *Etd*, *Slc9a2*, *Peg10*, *Pde4b* and *Aqp8* showed significant (P < 0.05) age-dependent differences between controls

To confirm the data from the RNAseq analysis 10 genes were chosen for further analysis by qPCR. These transcripts represent different levels of fold-change in expression after DTX and transcript levels were measured in testes from iDTR mice (without prior busulfan treatment) at different times after DTX treatment (Fig. 2). At 7 days after DTX treatment there was a clear and marked reduction in all transcript levels and the fold change after DTX was similar whether measured by qPCR or RNAseq with the exception of *Epha8* which showed a less marked reduction by qPCR. This confirms the reliability of data in Additional file 5 and shows that the changes in transcript levels after DTX are independent of previous exposure to busulfan. Expression levels of most of the Sertoli cell transcripts remained very low, and in some cased decreased further, up to 1 year after DTX treatment. In two interesting cases (*Etd* and *Defb36*) there was a transient increase in expression 30d after DTX treatment. This shows that other testicular cells had started expressing these transcripts at this time, a phenomenon that has been described previously following Leydig cell ablation [36]. Normal developmental changes in the same 10 Sertoli cell-specific transcripts are shown in Additional file 6 along with changes in the known Sertoli cell genes *Rhox5*, *Cldn11* and *Amh*. Most transcripts showed a developmental pattern similar to *Rhox5* (gradual developmental increase with more marked increase towards adulthood) or *Cldn11* (marked increase on day 10 with sustained high levels up to adulthood).

### Functional analysis of Sertoli cell-specific transcripts

Potential protein/gene network interactions among the Sertoli cell-specific transcripts identified by the online “Search Tool for the Retrieval of Interacting Genes/Proteins” (STRING, http://www.string-db.org) [37] are shown in Additional file 7. Functional analysis shows significant enrichment for 46 functional pathways within the set of Sertoli cell-specific transcripts, 20 cellular component pathways and 1 KEGG pathway (Additional file 8). A number of these enriched pathways/components are associated with known Sertoli cell specific functions such as junctional signalling and cell secretion and transport. It is also notable that the list of Sertoli cell specific transcripts includes 42 encoding different receptors, including VDR, FSHR, ADORA1, NR0B1 (DAX1) and AGTR2 (Additional file 9), while 111 of the translated proteins are predicted to have signal peptides and 72 may be secreted by the cells (Additional file 9). This list of 72 secreted proteins includes 10 which do not have a signal peptide and so must be secreted through non-classical secretory pathways [38]. Interestingly, the one KEGG pathway significantly enriched is for axon guidance while biological processes associated with neural signalling are also enriched. This is consistent with the reported ability of Sertoli cells to promote neuronal differentiation and survival in co-cultures and co-grafts [39] although the normal physiological importance of this is not clear beyond the high potential for the Sertoli cells to secrete trophic factors.

### Identification of germ cell-specific transcripts

In the same way that Sertoli cell specific genes have been identified by Sertoli cell ablation it is possible through a comparison between control testes and busulfan-treated testes to identify germ cell-specific transcripts. Busulfan acts by inducing apoptosis in spermatogonia within 1 week of treatment [40] and germ cells are then gradually lost from the testis over a 25–30 day period as remaining cells mature, are released from the testis and are not replaced. At the timepoint chosen for this study (50 days after busulfan) the testes are largely devoid of germ cells although some tubules contain small pockets of regenerating germ cells [17]. It is unlikely that these regenerating cells would have a marked effect on identification of germ cell genes given the small numbers but they may cause some genes (eg those highly expressed early in regeneration) to fall outside the criteria for selection. Selecting genes that show a significant >90% reduction in transcript levels after busulfan treatment identifies 4302 genes (Additional file 10) which includes a large number (2274) of uncharacterised genes. This is a fairly stringent approach and less stringent cutoffs can be applied to the data in Additional file 3. With the cutoff set at >90% reduction in transcript levels, it is highly likely that the vast majority of genes in Additional file 10 are of specific germ cell origin and the most highly expressed of these genes are the protamines and transition proteins. Changes in transcript levels of 4 selected germ cell-specific genes at different times after a single injection of busulfan are shown in Additional file 11. For 2 genes (*Nmur1* and *Chn2*) expression did not change until 30 days after busulfan injection indicating that these transcripts are expressed primarily in spermatids [17]. The remaining genes (*Sofu* and *Osbp2*) started to show a change in transcript levels around days 15–20 suggesting expression in spermatocytes.

Potential protein/gene network interactions among the germ cell-specific transcripts identified by STRING are shown in Additional file 12. Due to limitations on the number of genes/proteins that can be analysed by STRING only transcripts with average control expression >3 FPKM (2838 transcript species) were included, of which STRING recognised 1915 genes/proteins. Functional analysis shows significant enrichment for 36 functional pathways within the set of germ cell-specific transcripts, 35 cellular component pathways, 9 PFAM protein domains and 14 INTERPRO protein domains and features (Additional file 13). Most of these enriched pathways are associated with known germ cell-specific functions or features.

### Comparison to previously published Sertoli cell-specific gene lists

Previous studies have described the Sertoli cell transcriptome through the use of RiboTag mice or FACS [41–43] although there is little agreement between studies on what constitutes the Sertoli cell transcriptome. To determine whether there is a consensus between Sertoli cell-specific genes identified in this study and those identified by other groups using different methods we have mapped the genes from different studies to the results of the RNAseq (Additional file 14). Sanz et al. [41] identified 2430 Sertoli cell specific/enriched genes by RiboTag analysis and, of these, 448 show a 70% or greater decrease after DTX in our studies and 315 would satisfy the criteria of 2-fold increase after busulfan and 70% decrease after DTX (Additional file 14). De Gendt et al. [42] also used a RiboTag approach and identified 508 Sertoli cell-specific genes. Of these genes, 177 show a 70% decrease after DTX in our study and 122 would satisfy the criteria of 2-fold increase after busulfan and 70% decrease after DTX (Additional file 14). Using cell sorting techniques Zimmerman et al. [43] identified 8459 genes expressed in the Sertoli cell although this list is not Sertoli cell-specific; rather, it is a list of all genes expressed in the Sertoli cells and so contains genes expressed in other testicular cell types (eg *Actb* (β-actin)). Of the 495 protein-coding genes identified above as Sertoli cell-specific through DTX ablation (Additional file 5), 317 are also identified in the Sertoli cell list reported by Zimmerman et al. [43]. In contrast to protein-coding genes, only 18 of the non-coding/unclassified genes identified using DTX are also included in [43]. Comparing all 4 datasets (Additional file 5, two RiboTag datasets [41, 42] and the data in [43]) there are 83 transcripts that appear in all (Additional file 15). This may be considered as a baseline list of Sertoli cell-specific transcripts but must be considered to be highly conservative and treated with caution as it does not include some known Sertoli cell-specific transcripts such as *Rhox5*, *Amh*, *Sox9*, and *Gdnf*.

### Identification of FSH- and androgen-regulated Sertoli cell-specific transcripts

To identify Sertoli cell-specific genes regulated by FSH and androgen, microarrays were carried out using RNA extracted from 20-day old control, FSHRKO, SCARKO and FSHRKO.SCARKO mice. All data from the arrays is shown in Additional file 16 and data from Sertoli cell-specific transcripts only is shown in Additional file 17. In total, 539 Sertoli cell-specific transcripts, identified by RNAseq as above, were also present on the gene-chips used for the arrays.

**Fig. 3:**
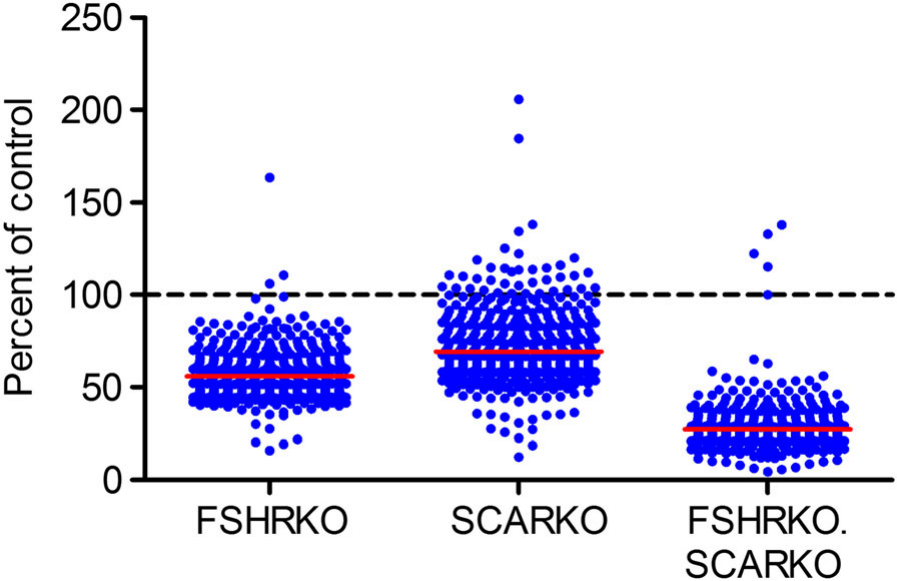
Summary of array data. Results show the change in expression (relative to control) of all Sertoli-cell specific transcripts in FSHRKO, SCARKO and FSHRKO.SCARKO mice at 20 days when normalised to Sertoli cell number. Each point represents a specific transcript species. The mean change of all transcripts in each group is shown as a red line

It has been shown previously that testis volume and Sertoli cell number differ significantly between the control and transgenic mouse groups used in this study [16] with the consequence that there is enrichment for Sertoli cell mRNA in total testicular mRNA from all groups relative to control animals (ie Sertoli cell mRNA will make up a greater proportion of total testis mRNA). To compare Sertoli cell transcript levels in the different mouse groups it is necessary, therefore, to normalise the array data to take into account testis volume and Sertoli cell number as previously described [44]. The Sertoli cell mRNA enrichment factor for each group was calculated as [Sertoli cell number in group X/testis volume group X]/[control Sertoli cell number/control volume] (where group X is FSHRKO, SCARKO or FSHRKO.SCARKO) using data from [16] (Additional file 1). Array data in Additional file 17 were normalised for Sertoli cell numbers by dividing by the enrichment factor (Additional file 1). Overall, the effects of the FSHR and the Sertoli cell AR knockouts was to decrease the level of most Sertoli cell-specific transcripts with a more marked effect in the double knockout (Fig. 3 and Additional file 17). More specifically, 510 Sertoli cell transcripts were significantly altered in the FSHRKO mouse and, of these, 509 were down regulated and only one (*Igfbp3*) significantly up regulated (Additional file 17). The most affected transcripts (*Emb*, *Dmrtc1b*, *Aqp8*) showed a 80–85% decrease in FSHRKO mice. In SCARKO mice 468 transcripts were significantly altered with 461 transcripts significantly decreased and 7 significantly increased. The greatest increase (2-fold) was in *Clca2* while the biggest decreases (80–90%) were in *Aqp8*, *Gm648* and *Corin*. Analysis showed that there was no significant interaction between the factors (FSHRKO and SCARKO) in 251 transcripts which means that any effects of FSHRKO and SCARKO were additive in the FSHRKO.SCARKO mouse. The significant interaction seen in 288 transcripts means that in the FSHRKO.SCARKO mouse the effect of the double knockout was not the predicted additive effects of the individual knockouts (ie there was either a significant synergistic effect of the double knockout or the double knockout was significantly less than the additive effects of the individual knockouts). In those transcripts showing a significant interaction there were 7 that showed a marked (>90%) decrease in the FSHRKO.SCARKO mice (*Emb*, *Aqp8*, *Dmrtc1b*, *Gm648*, *Nxf3*, *Corin*, *Slc9a2*) (Additional file 17). No transcript showed both an overall increase in the FSHRKO.SCARKO mice and a significant interaction between factors.

**Fig. 4:**
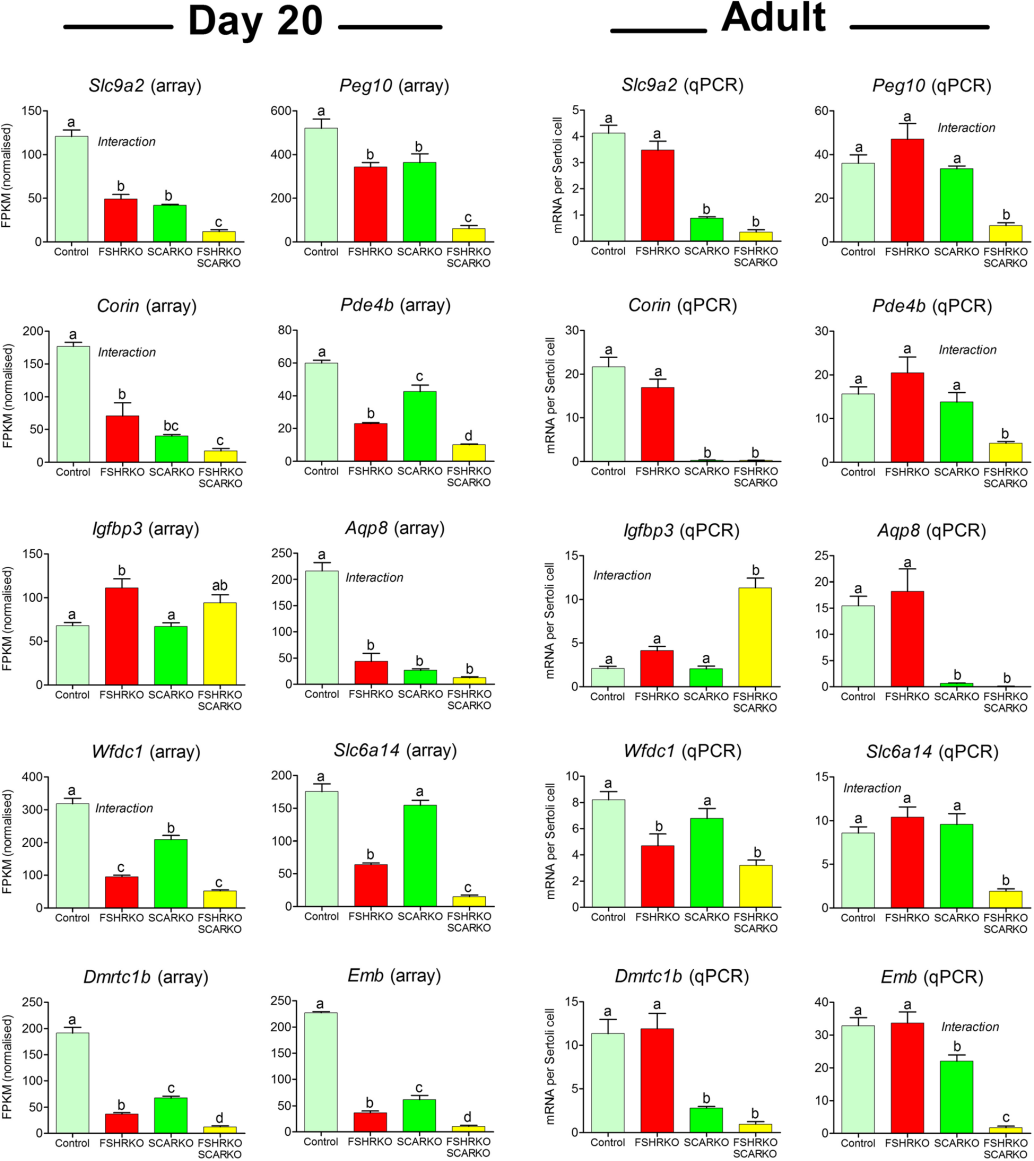
Selected transcript levels at day 20 and adulthood in FSHRKO, SCARKO and FSHRKO.SCARKO mice. Transcript levels were measured by RNAseq or qPCR as indicated and results show the mean ± SEM (*n* = 3–6 animals per group). Expression data has been normalised to Sertoli cell number for each group. Data was analysed initially by 2-factor ANOVA and then by Tukey’s post-hoc test. Where an interaction is indicated this means that the effect of the double knockout (FSHRKO.SCARKO) is significantly different from the additive effects of each individual knockout. Groups with different letter superscripts are significantly (P < 0.05) different

**Fig. 5:**
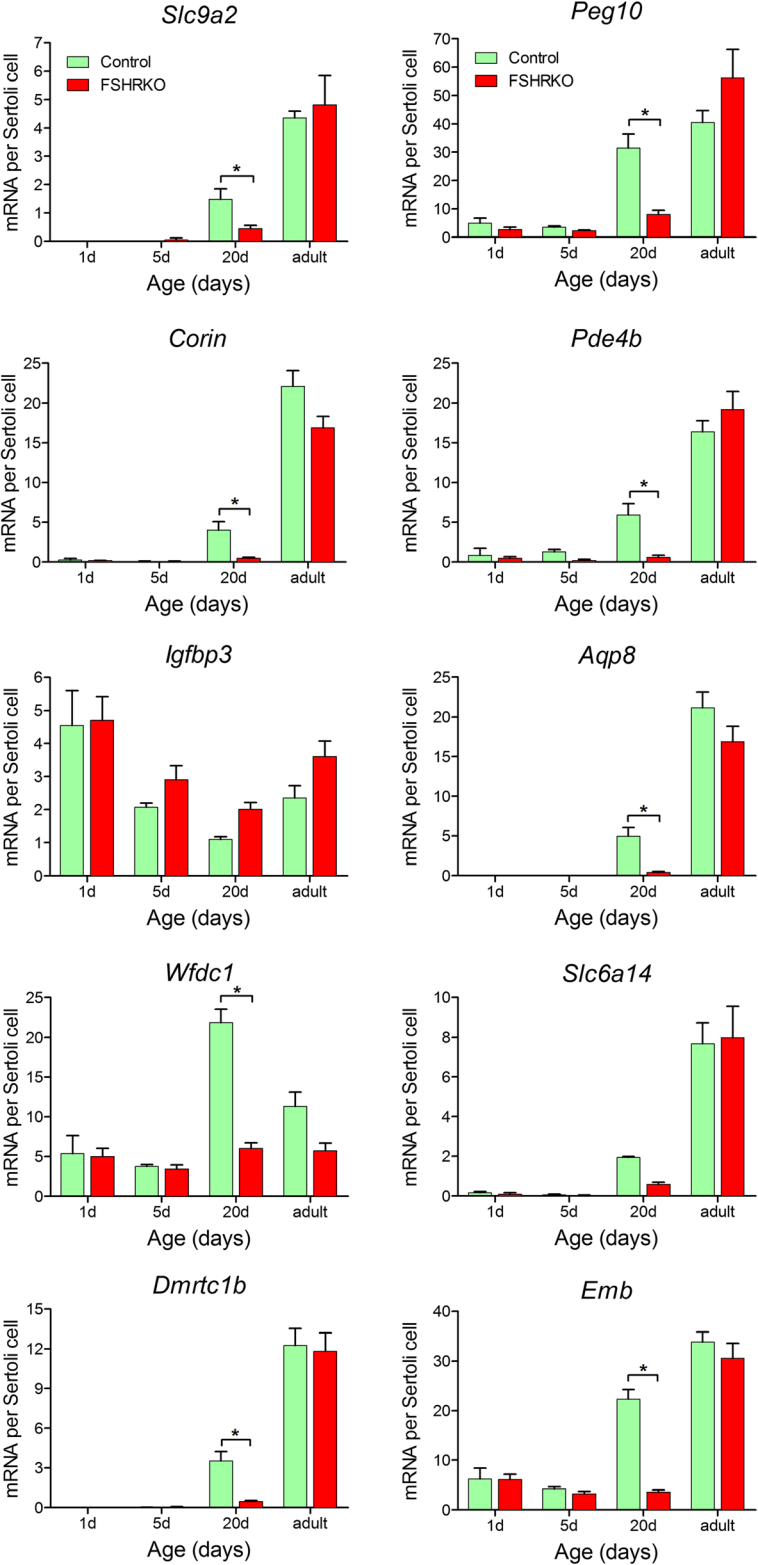
Developmental changes in transcript expression levels in control and FSHRKO mice measured by qPCR. Results show mean ± SEM of 3–6 animals per group. A significant (P < 0.05) difference between control and FSHRKO groups at a particular age is shown by an asterisk (*). Expression of *Igfbp3* and *Slc6a14* showed an overall effect of DTX but no individual ages showed significant effects

The array studies used 20-day old mice for reasons previously described [44]; briefly, this age was chosen because Sertoli cell proliferation has ceased and it is the first age at which Sertoli cells are likely to be showing an adult phenotype, it is prior to normal testicular descent and germ cell numbers have not yet reached adult levels (although the lack of more mature germ cells (eg spermatids) will alter cell signalling within the tubule). In order, therefore, to determine whether transcript expression in the adult shows similar dependence on hormonal stimulation to 20-day old animals, the expression of 10 Sertoli cell-specific genes was measured by qPCR in adult control, FSHRKO, SCARKO and FSHRKO.SCARKO mice. The transcripts chosen for further study represented a cross-section of transcript differences in the FSHRKO and SCARKO testes as measured by the microarrays. The qPCR used an external standard (luciferase) for normalisation so that there was no requirement to correct for testis volume and the results are corrected only for Sertoli cell number [16]. For comparison, data in Fig. 4 shows results from the arrays on day 20 animals and the qPCR studies on the adult animals. In general, transcript differences seen in adult SCARKO and FSHRKO.SCARKO mice were very similar to those seen at day 20 (Fig. 3). There was less agreement, however, in the FSHRKO mice with the effects of the knockout at day 20 more marked than in the adult. To confirm these differences we measured transcript expression in FSHRKO mice of different ages by qPCR (Fig. 5). Results show that the effects of the FSHR knockout are much greater at day 20 (as predicted by the arrays) than in the adult indicating that the Sertoli cells can largely adapt to loss of the FSHR in the adult.

## Discussion

The central role of the Sertoli cells in testis development and function is now established but how the Sertoli cells exert control over germ cells, Leydig cells and peritubular myoid cells remains largely unclear. Similarly, it remains uncertain how the Sertoli cells themselves are regulated by both endocrine and paracrine factors although progress has been made through use of FSHRKO and SCARKO mice, along with other mouse models. In this study, we have taken a significant step towards understanding both functional and control mechanisms associated with the Sertoli cell through identification of both the Sertoli cell-specific transcriptome and the regulation of that transcriptome by FSH and androgen.

The approach used here to identify Sertoli cell-specific transcripts has been used previously to identify the Leydig cell-specific transcriptome in the rat following EDS treatment [9, 45] and would be expected to identify the overwhelming majority of cell-specific transcripts with the same caveats described previously [9]. Firstly, transcripts could be erroneously designated as Sertoli cell-specific if Sertoli cell ablation caused a rapid and marked decline in transcript expression in another cell type. This is the main reason why a busulfan-treated, germ cell-free model was used for this study as Sertoli cell ablation will cause rapid loss of germ cells from the testis [2]. Nevertheless, it is possible that a small number of genes identified as Sertoli cell-specific may be Sertoli cell-dependent in another cell type. Secondly, any Sertoli cell-specific transcripts that are highly regulated by the germ cells and that are markedly down-regulated by germ cell ablation will not meet the stringency criteria set here and will be missing from the Sertoli cell-specific list. Thirdly, it is possible that Sertoli cell ablation could lead to the onset of expression of (normally) Sertoli cell-specific transcripts in another cell type. This has been described previously for a Leydig cell specific transcript following Leydig cell ablation [36] and the transient re-appearance of *Etd* and *Defb36* 30 days after Sertoli cell ablation (Fig. 2) suggests that this phenomenon may occur after Sertoli cell ablation. If similar ectopic expression of some transcripts occurs soon after Sertoli cell ablation it would mean that a small subset of Sertoli cell-specific transcripts may be missing from the list in Additional file 5. Finally, busulfan is a cytotoxic compound and it could have direct effects on the transcriptional regulation of somatic cells which would affect the data reported here. Mice do not normally express DTR and would not be expected to be sensitive to off-target effects of DTX but studies have shown that DTX can have receptor-independent effects including weight loss and cochlear damage [46]. It is possible, therefore, that DTX itself could also directly affect transcription in the non-target cells of the testis.

The stringency limits used here to define Sertoli cell-specific transcripts are largely arbitrary but were chosen to ensure that known Sertoli cell genes were not excluded. There was some variation in the residual expression of the Sertoli cell transcripts after Sertoli cell ablation (eg *Wt1*) which is a likely indication that some of these transcipts are also expressed to a small extent in another cell type. Alternatively, we have shown that Sertoli-like cells of the tubuli recti do not express AMH-Cre and are not ablated by DTX in the iDTR model [2, 3]. For most Sertoli cell-specific transcripts these cells contribute a low background level of expression following DTX-treatment but it is possible that genes such as *Wt1* are more highly expressed in the tubuli recti. Either way, Investigators can apply higher or lower stringency limits to the data in Additional file 3 as appropriate to other studies.

The Sertoli cell-specific gene list described here does not show strong similarity to other Sertoli cell-specific lists compiled using the RiboTag mouse with only 10–20% of transcripts matching [41, 42]. Perhaps surprisingly, the two RiboTag studies also show only 17% agreement between themselves. The Sertoli cell transcriptome described by Zimmermann et al. [43] is, however, largely consistent with data reported here. This transcriptome contains all transcript species expressed in the Sertoli cells and over 63% of the protein-coding genes identified here as Sertoli cell-specific are also included in the overall Sertoli cell transcriptome [43]. In addition, most of the remaining 37% of non-matching, Sertoli cell-specifc, protein-coding genes are expressed at low levels in control animals in our study (expression of 141 out of a total of 184 of these genes was <1 FPKM in controls) and may have fallen below the background expression cutoff used by Zimmermann et al. [43]. Comparing the datasets from the RiboTag mice [41, 42] with the overall Sertoli cell transcriptome [43] shows that 82% [41] and 71% [42] of genes identified in the RiboTag mice are expressed in the Sertoli cells, as may be expected, but the data outlined here using the iDTR mouse would suggest that the criteria for selecting Sertoli cell-specific/enriched genes may not have had adequate stringency.

Studies using a variety of techniques, including cell ablation, have highlighted the role that the Sertoli cells play in regulating testicular function in the adult [2, 3, 46–48]. These Sertoli cell actions include maintenance of germ cell and Leydig cell populations, stimulation of peritubular myoid cell activity and maintenance of the testicular vasculature [4]. How the Sertoli cells act to orchestrate each of these activities and maintain testicular function remains largely unknown, however. The identification, here, of 72 proteins/peptides that are Sertoli cell-specific and potentially secreted by the cells now provides a valuable resource for picking apart cell-cell interactions in the adult testis. The Sertoli cells also express 42 different receptor types which illustrates the complex variety of inputs possible for each cell. These receptors include some which have been previously reported to be expressed in the Sertoli cells and to play a role in testis development and adult function such as VDR and NR0B1 (DAX1) [49, 50]. Others, such as the type 2 angiotensin II receptor (AGTR2) have not previously been shown in the Sertoli cell but mean that the cells will be responsive to angiotensin. Previous studies have reported that angiotensinogen (*Agt*), renin (*Ren1*) and angiotensin converting enzyme (*Ace*) transcripts are all expressed in the Leydig cells and germ cells of the testis [51] (and consistent with the RNAseq data (Additional file 3) reported here). The testis is likely, therefore, to have an active renin-angiotensin system and this may be another mechanism by which the Leydig cells/germ cells can regulate Sertoli cell activity. The list of receptors also includes 11 olfactory receptors although transcript levels in the Sertoli cells are generally low and the function of ectopic olfactory receptors remains uncertain in many cases [52]. Nevertheless, the Sertoli cells also specifically express *Reep1* at high levels and REEP1 functions to enhance cell surface expression of odorant receptors [53] suggesting they may be functional in the Sertoli cells.

Development of the FSHRKO, SCARKO and combined FSHRKO.SCARKO mouse models has considerably enhanced our understanding of the endocrine control of Sertoli cell development, particularly with respect to cell proliferation and spermatogenesis [12–14, 16]. Studies into the control of Sertoli cell transcript levels have been more difficult to interpret, however, since these knockout mouse models have marked effects on germ cell numbers and a comprehensive, Sertoli cell-specific transcriptome has been missing. This has made it challenging to identify, beyond the limited number of previously known Sertoli cell-specific transcripts, which transcript changes in each model are due to altered Sertoli cell activity and which are due to a reduction in germ cell number. Identification of the Sertoli cell-specific transcriptome has allowed us now to examine the effects of the gene-specific knockouts on Sertoli cell activity. Results show that at 20 days of age in the mouse, shortly after Sertoli cell proliferation has ceased and as the blood-testis barrier is forming [54, 55], normal expression of most Sertoli cell-specific transcripts is dependent on both androgen and FSH with the effects of the two hormones either additive or synergistic. Given that the Sertoli cells regulate germ cell development and differentiation, this Sertoli cell dependence on hormonal stimulation around 20 days is consistent with earlier studies which showed that there is significant germ cell loss in both FSHRKO and SCARKO mice at 20d [16]. At 20 days the first wave of spermatogenesis is ongoing and it is characterised by a marked surge in germ cell apoptosis which is necessary for subsequent normal spermatogenesis [56]. Germ cell death during the first wave is regulated by FSH [57, 58] and, possibly, by androgen [59] and results described here show that this regulation of spermatogenesis is associated with changes in abundance of most Sertoli cell-specific transcripts.

Data from animals lacking the AR show that in the adult androgens remain essential for normal expression of most Sertoli cell-specific transcripts and in some cases (eg *Corin*) the requirement for androgen becomes more acute as the animals age. This is consistent with previous data showing that androgen stimulation through the Sertoli cells is essential for spermatogenesis in the adult testis [60]. In contrast, the role of FSH in adult testis biology has been uncertain for a number of years. Early studies showed that adult Sertoli cells in the rat or mouse are largely unresponsive to FSH [61] although FSH was thought to be required for the normal quantitative maintenance of germ cell production [62, 63]. Subsequent development of mice lacking FSH or its receptor (FSHR) showed that FSH is not required for fertility [12, 64] although adult germ cell numbers are reduced [14, 65], an effect which may be due to loss of FSH in the adult or to abnormal spermatogenesis during the first pubertal wave [66]. Results reported here now show that while FSH is essential for normal Sertoli cell transcript levels at puberty, cell activity in the adult mouse appears to be largely unaffected by loss of the FSHR. In some cases, however, the effect of losing both FSHR and AR from the Sertoli cells is more marked than loss of AR alone which indicates that, in the absence of androgen, FSH can act to regulate Sertoli cell activity in the adult but cannot overcome the phenotype associated with androgen deficiency.

## Conclusions

The Sertoli cells act to induce testis differentiation and subsequent development in fetal and post-natal life [2–6, 67] and they are essential for normal fertility in the adult. Further progress towards understanding the mechanisms which regulate these functions requires that we recognise how the different cell types in the testis are able to communicate. Identification of a robust Sertoli cell-specific transcriptome takes us closer to that goal and, combined with the total Sertoli cell transcriptome published earlier [43], provides us with the tools necessary to investigate regulation and function of the Sertoli cell. As an example of the potential offered by this data we have also shown, using microarray data, that most Sertoli cell-specific transcripts are FSH- and androgen-dependent during development and that they remain androgen-dependent into adulthood. This is likely to explain the observation that androgen action through the Sertoli cells is essential for spermatocyte progression through meiosis [60] and suggests that the effect of androgen may be a general increase in cell activity rather than activation of specific pathways.

## Additional files

Additional file 1: “Correction factors for array data”. This document contains data on testis volume and Sertoli cell number used to normalise array data. (DOCX 13 kb)

Additional file 2: “Primers used for real-time PCR”. (XLSX 11 kb)

Additional file 3: “Total RNAseq data.” All RNAseq data from control, busulfan-treated and busulfan + DTX-treated mice. (XLSX 5119 kb)

Additional file 4: “Transcripts significantly altered by busulfan and/or busulfan + DTX.” List of transcripts significantly altered in the RNAseq data following treatment with busulfan or busulfan + DTX. (XLSX 960 kb)

Additional file 5: “Sertoli cell-specific transcripts in mouse testis.” List of all Sertoli cell-specific transcripts identified using the criteria outlined in the text. (XLSX 195 kb)

Additional file 6: “Developmental changes in selected Sertoli cell-specific transcripts”. (PPTX 494 kb)

Additional file 7: “Protein interaction networks associated with mouse Sertoli cell-specific transcripts”. (PPTX 716 kb)

Additional file 8: “Functional pathways associated with Sertoli cell-specific transcripts.” List of pathways functionally enriched in the protein interaction network shown in Additional file 7. (XLSX 20 kb)

Additional file 9: “Sertoli cell-specific transcripts containing a signal peptide, encoding a putatively secreted protein or encoding a receptor”. (XLSX 19 kb)

Additional file 10: “Germ cell-specific transcripts in mouse testis.” List of all germ cell-specific transcripts identified using the criteria outlined in the text. (XLSX 1032 kb)

Additional file 11: “Changes in selected germ cell transcript levels following a single injection with busulfan.” Data shows effect of busulfan on germ cell-specific transcripts in normal mice. (PPTX 172 kb)

Additional file 12: “Protein interaction networks associated with mouse germ cell-specific transcripts”. (PPTX 729 kb)

Additional file 13: “Functional pathways associated with germ cell-specific transcripts.” List of pathways functionally enriched in the protein interaction network shown in Additional file 11. (XLSX 21 kb)

Additional file 14: “RNAseq data (from Additional file 3) showing expression of Sertoli cell transcripts identified by Sanz et al. or de Gendt et al.” Effect of busulfan and busulfan +DTX on transcripts identified by Sanz et al. [41] or de Gendt et al. [42] as Sertoli cell specific. (XLSX 651 kb)

Additional file 15: “Transcripts present in all databases of Sertoli cell transcripts.” Transcripts present in all the Sertoli cell-specific databases (this publication and [41, 42]) and in the total Sertoli cell transcriptome [43]. (XLSX 10 kb)

Additional file 16: “Array data from testes of 20-day old normal, FSHRKO, SCARKO and FSHRKO.SCARKO mice.” All array data from 4 animals in each goup. (XLSX 5902 kb)

Additional file 17: “Array data showing changes in Sertoli cell-specific transcripts in FSHRKO, SCARKO and FSHRKO.SCARKO mice.” Expression of Sertoli cell-specific transcripts, indentified in Additional file 5, on arrays of normal and knockout mice. Data has been normalised to Sertoli cell number and testis volume as described in the text. Data was analyses by two factor ANOVA with the FDR set at 0.05. (XLSX 136 kb)

Additional file 18: “ENA ids”. (XLSX 10 kb)

## References

[1] Chojnacka K, Zarzycka M, Mruk DD (2016). Biology of the Sertoli cell in the fetal, pubertal, and adult mammalian testis. Results Probl Cell Differ.

[2] Rebourcet D, O'Shaughnessy PJ, Monteiro A, Milne L, Cruickshanks L, Jeffrey N, Guillou F, Freeman TC, Mitchell RT, Smith LB (2014). Sertoli cells maintain Leydig cell number and peritubular myoid cell activity in the adult mouse testis. PLoS One.

[3] Rebourcet D, O'Shaughnessy PJ, Pitetti JL, Monteiro A, O'Hara L, Milne L, Tsai YT, Cruickshanks L, Riethmacher D, Guillou F, Mitchell RT, van't Hof R, Freeman TC, Nef S, Smith LB (2014). Sertoli cells control peritubular myoid cell fate and support adult Leydig cell development in the prepubertal testis. Development.

[4] Rebourcet D, Wu J, Cruickshanks L, Smith SE, Milne L, Fernando A, Wallace RJ, Gray CD, Hadoke PW, Mitchell RT, O'Shaughnessy PJ, Smith LB (2016). Sertoli cells modulate testicular vascular network development, structure, and function to influence circulating testosterone concentrations in adult male mice. Endocrinology.

[5] Yao HH, Whoriskey W, Capel B (2002). Desert hedgehog/patched 1 signaling specifies fetal Leydig cell fate in testis organogenesis. Genes Dev.

[6] Bitgood MJ, Shen L, McMahon AP (1996). Sertoli cell signaling by desert hedgehog regulates the male germline. Curr Biol.

[7] Kloner RA, Carson C, Dobs A, Kopecky S, Mohler ER (2016). Testosterone and cardiovascular disease. J Am Coll Cardiol.

[8] Yeap BB, Araujo AB, Wittert GA (2012). Do low testosterone levels contribute to ill-health during male ageing?. Crit Rev Clin Lab Sci.

[9] O'Shaughnessy PJ, Monteiro A, Fowler PA, Morris ID (2014). Identification of Leydig cell-specific mRNA transcripts in the adult rat testis. Reproduction.

[10] Smith LB, Walker WH. Hormone signalling in the testis. In Knobil and Neill's physiology of reproduction. 4th edition. Edited by plant TM, Zeleznick AJ. Amsterdam: Elsevier. 2015:637–90.

[11] Krishnamurthy H, Danilovich N, Morales CR, Sairam MR (2000). Qualitative and quantitative decline in spermatogenesis of the follicle-stimulating hormone receptor knockout (FORKO) mouse. Biol Reprod.

[12] Abel MH, Wootton AN, Wilkins V, Huhtaniemi I, Knight PG, Charlton HM (2000). The effect of a null mutation in the follicle-stimulating hormone receptor gene on mouse reproduction. Endocrinology.

[13] De Gendt K, Swinnen JV, Saunders PT, Schoonjans L, Dewerchin M, Devos A, Tan K, Atanassova N, Claessens F, Lecureuil C, Heyns W, Carmeliet P, Guillou F, Sharpe RM, Verhoeven G (2004). A Sertoli cell-selective knockout of the androgen receptor causes spermatogenic arrest in meiosis. Proc Natl Acad Sci U S A.

[14] Abel MH, Baker PJ, Charlton HM, Monteiro A, Verhoeven G, De Gendt K, Guillou F, O'Shaughnessy PJ (2008). Spermatogenesis and sertoli cell activity in mice lacking sertoli cell receptors for follicle-stimulating hormone and androgen. Endocrinology.

[15] Verhoeven G, Willems A, Denolet E, Swinnen JV, De Gendt K (2010). Androgens and spermatogenesis: lessons from transgenic mouse models. Philos Trans R Soc Lond Ser B Biol Sci.

[16] O'Shaughnessy PJ, Monteiro A, Abel M (2012). Testicular development in mice lacking receptors for follicle stimulating hormone and androgen. PLoS One.

[17] O'Shaughnessy PJ, Hu L, Baker PJ (2008). Effect of germ cell depletion on levels of specific mRNA transcripts in mouse Sertoli cells and Leydig cells. Reproduction.

[18] O'Shaughnessy PJ, Willerton L, Baker PJ (2002). Changes in Leydig cell gene expression during development in the mouse. Biol Reprod.

[19] Martin M (2011). Cutadapt removes adapter sequences from high-throughput sequencing reads. EMBnet J.

[20] Kim D, Langmead B, Salzberg SLHISAT (2015). A fast spliced aligner with low memory requirements. Nat Methods.

[21] Trapnell C, Hendrickson DG, Sauvageau M, Goff L, Rinn JL, Pachter L (2013). Differential analysis of gene regulation at transcript resolution with RNA-seq. Nat Biotechnol.

[22] Trapnell C, Williams BA, Pertea G, Mortazavi A, Kwan G, van Baren MJ, Salzberg SL, Wold BJ, Pachter L (2010). Transcript assembly and quantification by RNA-Seq reveals unannotated transcripts and isoform switching during cell differentiation. Nat Biotechnol.

[23] Goff L, Trapnell C, Kelley D. cummeRbund: Analysis, exploration, manipulation and visualization of Cufflinks high-throughput sequencing data. 2012; R package version 2.18.0.

[24] Baban D, Davies KE (2008). Microarray analysis of mdx mice expressing high levels of utrophin: therapeutic implications for dystrophin deficiency. Neuromuscul Disord.

[25] O'Shaughnessy PJ, Murphy L (1993). Cytochrome P-450 17α-hydroxylase protein and mRNA in the testis of the testicular feminized (Tfm) mouse. J Mol Endocrinol.

[26] Baker PJ, O'Shaughnessy PJ (2001). Expression of prostaglandin D synthetase during development in the mouse testis. Reproduction.

[27] Fowler PA, Flannigan S, Mathers A, Gillanders K, Lea RG, Wood MJ, Maheshwari A, Bhattacharya S, Collie-Duguid ES, Baker PJ, Monteiro A, O'Shaughnessy PJ (2009). Gene expression analysis of human fetal ovarian primordial follicle formation. J Clin Endocrinol Metab.

[28] Czechowski T, Bari RP, Stitt M, Scheible WR, Udvardi MK (2004). Real-time RT-PCR profiling of over 1400 Arabidopsis transcription factors: unprecedented sensitivity reveals novel root- and shoot-specific genes. Plant J.

[29] O'Shaughnessy PJ, Monteiro A, Fowler PA (2011). Identification of stable endogenous reference genes for real-time PCR in the human fetal gonad using an external standard technique. Mol Hum Reprod.

[30] Benjamini Y, Hochberg Y. Controlling the false disovery rate: a practical and powerful approach to multiple testing. J R Statist Soc. 1995;57:289–300.

[31] Basciani S, Mariani S, Spera G, Gnessi L. Role of platelet-derived growth factors in the testis. Endocr Rev. 2010;31:916–39. 10.1210/er.2010-0004 20650860

[32] Wijayarathna R, de Kretser DM (2016). Activins in reproductive biology and beyond. Hum Reprod Update.

[33] Chalmel F, Lardenois A, Evrard B, Rolland AD, Sallou O, Dumargne MC, Coiffec I, Collin O, Primig M, Jégou B (2014). High-resolution profiling of novel transcribed regions during rat spermatogenesis. Biol Reprod.

[34] Chocu S, Evrard B, Lavigne R, Rolland AD, Aubry F, Jégou B, Chalmel F, Pineau C (2014). Forty-four novel protein-coding loci discovered using a proteomics informed by transcriptomics (PIT) approach in rat male germ cells. Biol Reprod.

[35] Daigle M, Roumaud P, Martin LJ (2015). Expressions of Sox9, Sox5, and Sox13 transcription factors in mice testis during postnatal development. Mol Cell Biochem.

[36] Meinhardt A, Bacher M, O'Bryan M, McFarlane J, Mallidis C, Lehmann CMC, de Kretser D, Bucala R, Hedger MA (1999). Switch in the cellular localization of macrophage migration inhibitory factor in the rat testis after ethane dimethane sulphonate treatment. J cell. Science.

[37] Szklarczyk D, Franceschini A, Wyder S, Forslund K, Heller D, Huerta-Cepas J, Simonovic M, Roth A, Santos A, Tsafou KP, Kuhn M, Bork P, Jensen LJ, STRING v MC (2015). v10: protein-protein interaction networks, integrated over the tree of life. Nucleic Acids Res.

[38] Chua CE, Lim YS, Lee MG, Tang BL (2012). Non-classical membrane trafficking processes galore. J Cell Physiol.

[39] Deng L, Shi B, Zhuang Y, Chu J, Shi X, Zhang S, Guo M (2014). Performance and mechanism of neuroleukin in the growth and survival of sertoli cell-induced neurons in a coculture system. Cell Transplant.

[40] Choi YJ, Ok DW, Kwon DN, Chung JI, Kim HC, Yeo SM, Kim T, Seo HG, Kim JH (2004). Murine male germ cell apoptosis induced by busulfan treatment correlates with loss of c-kit-expression in a Fas/FasL- and p53-independent manner. FEBS Lett.

[41] Sanz E, Evanoff R, Quintana A, Evans E, Miller JA, Ko C, Amieux PS, Griswold MD, McKnight GS (2013). RiboTag analysis of actively translated mRNAs in Sertoli and Leydig cells in vivo. PLoS One.

[42] De Gendt K, Verhoeven G, Amieux PS, Wilkinson MF (2014). Genome-wide identification of AR-regulated genes translated in Sertoli cells in vivo using the RiboTag approach. Mol Endocrinol.

[43] Zimmermann C, Stevant I, Borel C, Conne B, Pitetti JL, Calvel P, Kaessmann H, Jegou B, Chalmel F, Nef S (2015). Research resource: the dynamic transcriptional profile of sertoli cells during the progression of spermatogenesis. Mol Endocrinol.

[44] O'Shaughnessy PJ, Abel M, Charlton HM, Hu B, Johnston H, Baker PJ (2007). Altered expression of genes involved in regulation of vitamin a metabolism, solute transportation, and cytoskeletal function in the androgen-insensitive tfm mouse testis. Endocrinology.

[45] Zhang YF, Yuan KM, Liang Y, Chu YH, Lian QQ, Ge YF, Zhen W, Sottas CM, ZJ S, Ge RS (2015). Alterations of gene profiles in Leydig-cell-regenerating adult rat testis after ethane dimethane sulfonate-treatment. Asian J Androl.

[46] Konishi H, Ohgami N, Matsushita A, Kondo Y, Aoyama Y, Kobayashi M, Nagai T, Ugawa S, Yamada K, Kato M, Kiyama H (2017). Exposure to diphtheria toxin during the juvenile period impairs both inner and outer hair cells in C57BL/6 mice. Neuroscience.

[47] Mruk DD, Cheng CY (2015). The mammalian blood-testis barrier: its biology and regulation. Endocr Rev.

[48] Franca LR, Hess RA, Dufour JM, Hofmann MC, Griswold MD (2016). The Sertoli cell: one hundred fifty years of beauty and plasticity. Andrology.

[49] Kinuta K, Tanaka H, Moriwake T, Aya K, Kato S, Seino Y, Vitamin D (2000). Is an important factor in estrogen biosynthesis of both female and male gonads. Endocrinology.

[50] Park SY, Lee EJ, Emge D, Jahn CL, Jameson JLA (2008). Phenotypic spectrum of sexual development in Dax1 (Nr0b1)-deficient mice: consequence of the C57BL/6J strain on sex determination. Biol Reprod.

[51] Hayden R, Tanrikut C (2014). Manipulation of a locally expressed renin-angiotensin system in the testis: implications for steroidogenesis. J Urol.

[52] Kng N, Koo J (2012). Olfactory receptors in non-chemosensory tissues. BMB Rep.

[53] Saito H, Kubota M, Roberts RW, Chi Q, Matsunami HRTP (2004). Family members induce functional expression of mammalian odorant receptors. Cell.

[54] Baker PJ, O'Shaughnessy PJ (2001). Role of gonadotrophins in regulating numbers of Leydig and Sertoli cells during fetal and postnatal development in mice. Reproduction.

[55] Hosoi I, Toyama Y, Maekawa M, Ito H, Yuasa S (2002). Development of the blood-testis barrier in the mouse is delayed by neonatally administered diethylstilbestrol but not by beta-estradiol 3-benzoate. Andrologia.

[56] Rodriguez I, Ody C, Araki K, Garcia I, Vassalli P (1997). An early and massive wave of germinal cell apoptosis is required for the development of functional spermatogenesis. EMBO J.

[57] Billig H, Furuta I, Rivier C, Tapanainen J, Parvinen M, Hsueh AJ (1995). Apoptosis in testis germ cells: developmental changes in gonadotropin dependence and localization to selective tubule stages. Endocrinology.

[58] Meachem SJ, Ruwanpura SM, Ziolkowski J, Ague JM, Skinner MK, Loveland KL (2005). Developmentally distinct in vivo effects of FSH on proliferation and apoptosis during testis maturation. J Endocrinol.

[59] Walczak-Jedrzejowska R, Kula K, Oszukowska E, Marchlewska K, Kula W, Slowikowska-Hilczer J (2011). Testosterone and oestradiol in concert protect seminiferous tubule maturation against inhibition by GnRH-antagonist. Int J Androl.

[60] O'Shaughnessy PJ, Verhoeven G, De Gendt K, Monteiro A, Abel MH (2010). Direct action through the sertoli cells is essential for androgen stimulation of spermatogenesis. Endocrinology.

[61] Means AR, Dedman JR, Tash JS, Tindall DJ, van Sickle M, Welsh MJ (1980). Regulation of the testis sertoli cell by follicle stimulating hormone. Annu Rev Physiol.

[62] Griswold MD, Russell LD, Griswold MD (1993). Action of FSH on mammalial Sertoli cells. The Sertoli cell.

[63] Zirkin BR, Awoniyi C, Griswold MD, Russell LD, Sharpe R (1994). Is FSH required for adult spermatogenesis?. J Androl.

[64] Kumar TR, Wang Y, Lu N, Matzuk MM (1997). Follicle stimulating hormone is required for ovarian follicle maturation but not male fertility. Nat Genet.

[65] Wreford NG, Rajendra Kumar T, Matzuk MM, de Kretser DM (2001). Analysis of the testicular phenotype of the follicle-stimulating hormone beta-subunit knockout and the activin type II receptor knockout mice by stereological analysis. Endocrinology.

[66] O'Shaughnessy PJ (2014). Hormonal control of germ cell development and spermatogenesis. Semin Cell Dev Biol.

[67] Palmer SJ, Burgoyne PS (1991). Situ analysis of fetal, prepuberal and adult XX/Xy chimeric mouse testes - sertoli cells are predominantly, but not exclusively, XY. Development.

