# Supplementary material for: Identification of Sertoli cell-specific transcripts in the mouse testis and the role of FSH and androgen in the control of Sertoli cell activity
Source: BMC Genomics. 2017 Dec 15;18:972. doi: 10.1186/s12864-017-4357-3 (PMC5731206; doi:10.1186/s12864-017-4357-3)
Supplement: Supplementary file 1 — “Correction factors for array data”. This document contains data on testis volume and Sertoli cell number used to normalise array data. (DOCX 13 kb) [file 12864_2017_4357_MOESM1_ESM.docx]

Correction factors for array data*

Group Testis volume (mm^3^) Sertoli cell number(x10^-5^) Enrichment factor^+^ Control 22.03 26.85 1

FSHRKO 8.87 22.94 2.12

SCARKO 13.95 29.70 1.74

FSHRKO. SCARKO 4.22 22.78 4.42

* Data from reference [16].

^+^Enrichment factor for each group is [Sertoli cell number/testis volume]/[control Sertoli cell number/control volume]
