# Supplementary material for: Identification of Sertoli cell-specific transcripts in the mouse testis and the role of FSH and androgen in the control of Sertoli cell activity
Source: BMC Genomics. 2017 Dec 15;18:972. doi: 10.1186/s12864-017-4357-3 (PMC5731206; doi:10.1186/s12864-017-4357-3)
Supplement: Supplementary file 6 — “Developmental changes in selected Sertoli cell-specific transcripts”. (PPTX 494 kb) [file 12864_2017_4357_MOESM6_ESM.pptx]

## Slide 1
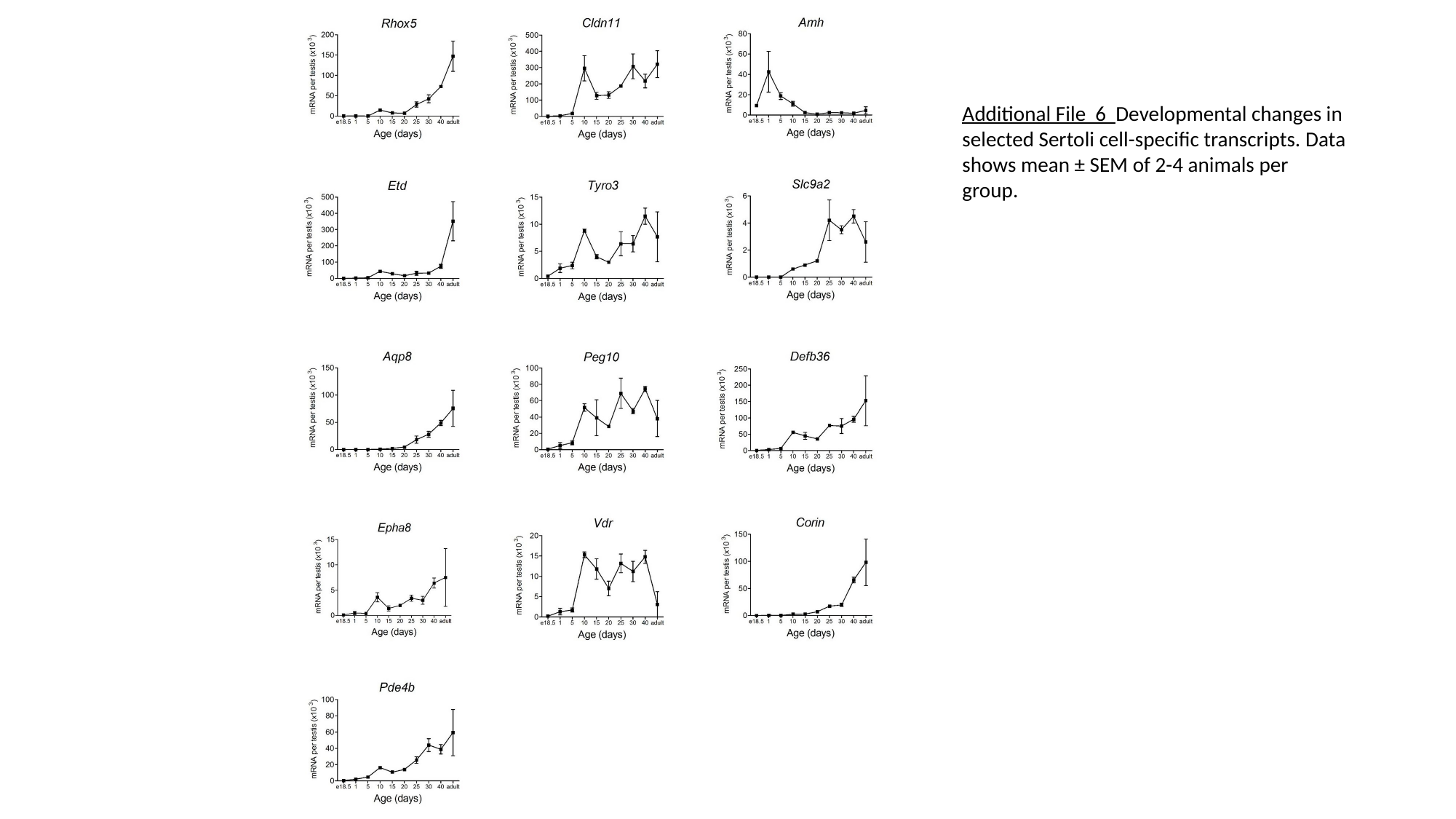

Additional File 6 Developmental changes in selected Sertoli cell-specific transcripts. Data shows mean ± SEM of 2-4 animals per group.
