# Supplementary material for: Identification of Sertoli cell-specific transcripts in the mouse testis and the role of FSH and androgen in the control of Sertoli cell activity
Source: BMC Genomics. 2017 Dec 15;18:972. doi: 10.1186/s12864-017-4357-3 (PMC5731206; doi:10.1186/s12864-017-4357-3)
Supplement: Supplementary file 7 — “Protein interaction networks associated with mouse Sertoli cell-specific transcripts”. (PPTX 716 kb) [file 12864_2017_4357_MOESM7_ESM.pptx]

## Slide 1
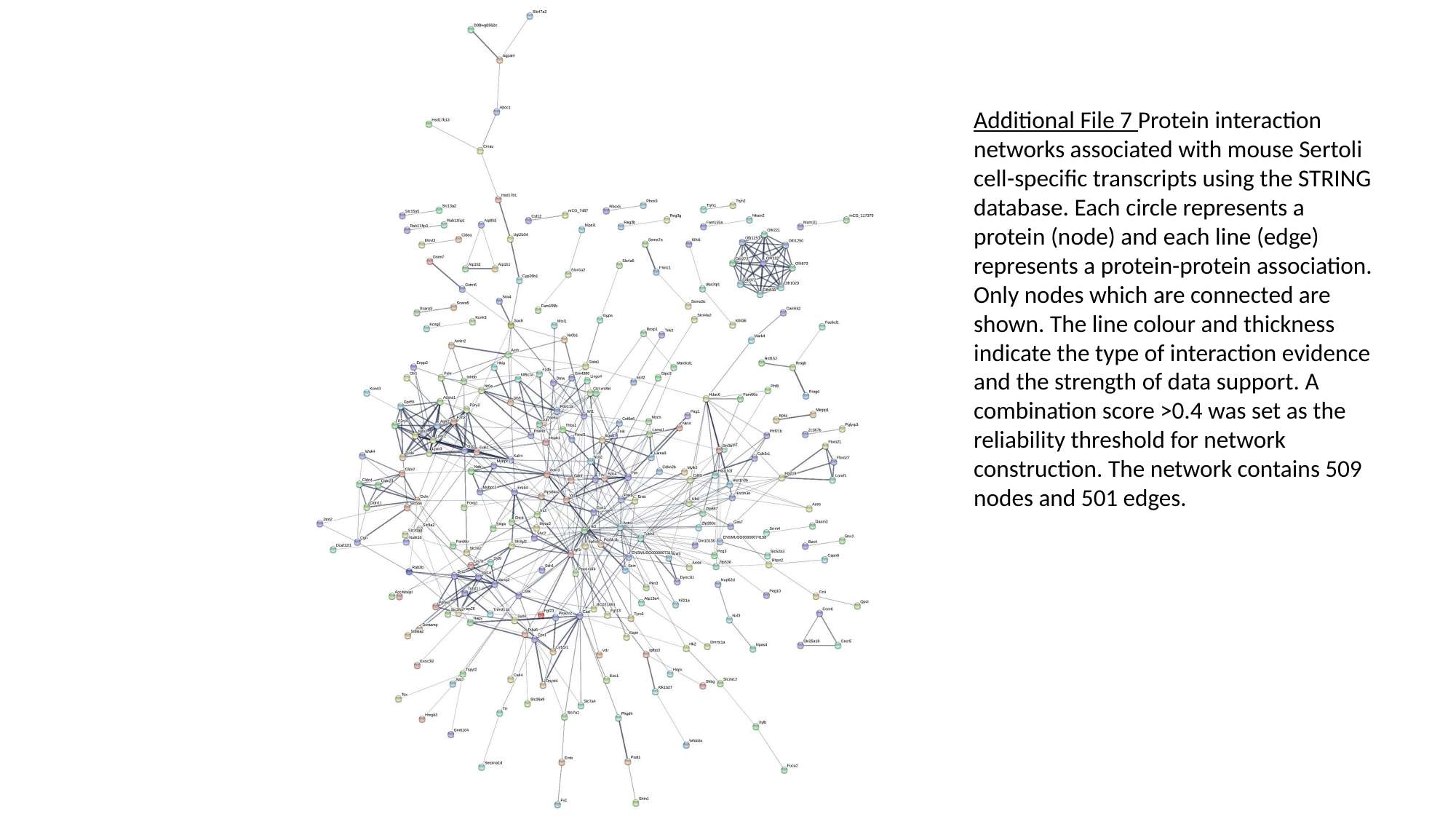

Additional File 7 Protein interaction networks associated with mouse Sertoli cell-specific transcripts using the STRING database. Each circle represents a protein (node) and each line (edge) represents a protein-protein association. Only nodes which are connected are shown. The line colour and thickness indicate the type of interaction evidence and the strength of data support. A combination score >0.4 was set as the reliability threshold for network construction. The network contains 509 nodes and 501 edges.
