# Supplementary material for: Identification of Sertoli cell-specific transcripts in the mouse testis and the role of FSH and androgen in the control of Sertoli cell activity
Source: BMC Genomics. 2017 Dec 15;18:972. doi: 10.1186/s12864-017-4357-3 (PMC5731206; doi:10.1186/s12864-017-4357-3)
Supplement: Supplementary file 11 — “Changes in selected germ cell transcript levels following a single injection with busulfan.” Data shows effect of busulfan on germ cell-specific transcripts in normal mice. (PPTX 172 kb) [file 12864_2017_4357_MOESM11_ESM.pptx]

## Slide 1
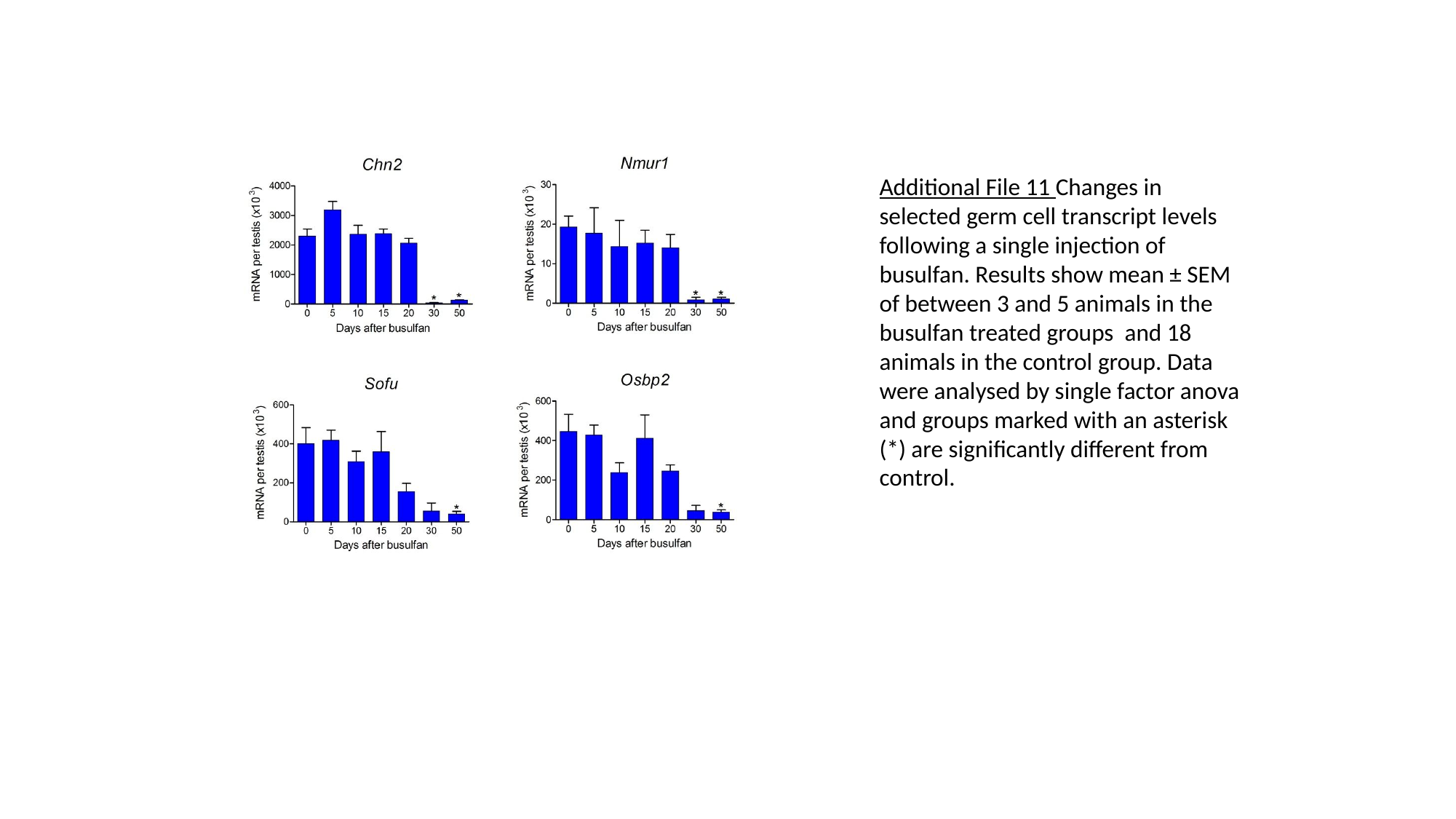

Additional File 11 Changes in selected germ cell transcript levels following a single injection of busulfan. Results show mean ± SEM of between 3 and 5 animals in the busulfan treated groups and 18 animals in the control group. Data were analysed by single factor anova and groups marked with an asterisk (*) are significantly different from control.
